# Does competition improve financial stability of the banking sector in ASEAN countries? An empirical analysis

**DOI:** 10.1371/journal.pone.0176546

**Published:** 2017-05-09

**Authors:** Abu Hanifa Md. Noman, Chan Sok Gee, Che Ruhana Isa

**Affiliations:** 1 Department of Finance and Banking, Faculty of Business and Accountancy, University of Malaya, Kuala Lumpur, Malaysia; 2 Department of Business Administration, Faculty of Business Studies, International Islamic University Chittagong, Chittagong, Bangladesh; 3 Department of Accountancy, Faculty of Business and Accountancy, University of Malaya, Kuala Lumpur, Malaysia; Universidad Veracruzana, MEXICO

## Abstract

This study examines the influence of competition on the financial stability of the commercial banks of Association of Southeast Asian Nation (ASEAN) over the 1990 to 2014 period. Panzar-Rosse H-statistic, Lerner index and Herfindahl-Hirschman Index (HHI) are used as measures of competition, while Z-score, non-performing loan (NPL) ratio and equity ratio are used as measures of financial stability. Two-step system Generalized Method of Moments (GMM) estimates demonstrate that competition measured by H-statistic is positively related to Z-score and equity ratio, and negatively related to non-performing loan ratio. Conversely, market power measured by Lerner index is negatively related to Z-score and equity ratio and positively related to NPL ratio. These results strongly support the competition-stability view for ASEAN banks. We also capture the non-linear relationship between competition and financial stability by incorporating a quadratic term of competition in our models. The results show that the coefficient of the quadratic term of H-statistic is negative for the Z-score model given a positive coefficient of the linear term in the same model. These results support the non-linear relationship between competition and financial stability of the banking sector. The study contains significant policy implications for improving the financial stability of the commercial banks.

## Introduction

The effect of banking competition on financial stability has been an issue of active debate in academic and policy circles. This debate intensified in the aftermath of the 2008–09 global financial crisis (GFC) with growing concern among policy makers and academics regarding the extent to which competition is responsible for the crisis, while many banks failed and others lost their profitability and required additional capitalisation. Despite seeing competition as a pre-condition for efficiency, technological innovation, institutional development, and financial inclusion [1–3], there has been no consensus as to whether high competition leads to financial stability in the banking system.

Financial liberalisation in both matured and emerging economies since the late 1970s and early 1980s has increased competition in the banking sector which influenced large banks from matured countries operating at low-profit margins to penetrate emerging countries with a relatively high profit margin. Increased competition drives banking institutions to accelerate the consolidation process to protect their market power, which again raises concerns of increasing the number of large banks, and the level of concentration. In fact, the incidence of numerous financial crises in both matured and emerging economies in the last three decades and the resulting regulatory failures to bring the banking system in discipline have raised concerns among policy makers and academics regarding the subsequent effect of competition on financial stability in the banking system.

The relationship between competition and financial stability is ambiguous in theoretical predictions. The traditional competition-fragility view of Keeley [4] claims that excessive competition in the banking market erodes market power and profit margin of banks, and drives them to take high risk which is the cause of bank failure and instability in the banking market. Conversely, the modern competition-stability view of Boyd and Nicolo [5] claims that excessive competition in the banking market drives the banks to lower the loan interest rate which reduces moral hazard and adverse selection problem of the banks, reduces their default risk, and enhances financial stability. On the other hand, Martinez-Miera and Repullo [6] claim that both the competition-fragility view and competition-stability view can coexist, and the relationship between competition and financial stability is non-linear or inverted U-shaped.

For significant policy formulation, the nexus between competition and financial stability is investigated empirically focusing in both matured and emerging countries. However, the findings conclude with conflicting empirical results keeping the nexus between competition and financial stability a puzzle. This study investigates the effect of competition on the financial stability of commercial banks in the emerging economies of Association of Southeast Asian Nation (ASEAN). Further, it examines the effect of financial crisis on the competition-stability nexus that may be impaired by the crisis. Crisis may lead the banking sector to adopt different reform strategies, such as capital regulations, activity restrictions, and consolidation that may change the market power and risk-taking behaviour of the banks.

The ASEAN region provides a fertile laboratory to investigate the relationship between competition and financial stability because its banking sector has experienced liberalisation via foreign bank penetration in the early 1990s, followed by deregulation, regional economic integration, and tremendous consolidation in the late 1990s as post 1997–98 Asian financial crisis bank restructuring strategies. Further, its banking market is distinctive for at least two reasons. Firstly, ASEAN’s central banks pushed commercial banks towards consolidation to attain financial stability. Secondly, the governors of ASEAN central banks endorsed the ASEAN Banking Integration Framework (ABIF) in 2011 which will be implemented initially among ASEAN-5 countries including Indonesia, Malaysia, the Philippines, Singapore and Thailand by 2020. ABIF may increase competition and lead the banking market to increase efficiency and attain economy of scale [7]. Yet, the resulting increased competition in the regional banking market may push small banks towards further consolidation in order to strength their domestic presence and better competition with regional banks [8]. This initiative towards high concentration may allow regional banks to enjoy high monopoly power which is an issue of concern for policy makers and bank regulators, because high monopoly power leads to increased loan interest rates which undermine easy access to credit and financial inclusion, and put financial stability of the region at risk due to the high risk-taking tendency of the banks. Thus, the failure of one bank may quickly spill-over to others in the region and may cause another Asian financial crisis due to the tight knit regional banking system.

This study contributes to the literature in several ways. Firstly, it contributes to the debate on the competition-stability/fragility nexus by providing a new evidence from emerging ASEAN-5 countries. Secondly, it uses a long panel data covering 25 years ranging from 1990 to 2014 which captures early 1990s financial liberalization, both the 1997–98 AFC and 2008–09 GFC, post AFC deregulation and bank restructuring efforts. This allows us to develop an extensive database to capture competition and financial stability. To the best of our knowledge, all the relevant literature that examines the nexus between competition and financial stability are based on pre-global financial crisis except the work of Fu, Lin [9], which was limited to the 2008–09 GFC. Thirdly, the study investigates the effect of recent banking crises on the competition-stability relationship to diversify policy implications regarding consolidation, liberalisation, capitalisation, and activity restrictions. Fourthly, the study adds regulatory variables such as activity restrictions and deposit insurance in the econometric specification since they impair the relationship between competition and stability [10]. Finally, the study controls the possible endogeneity between competition and financial stability using two-step system Generalized Method of Moments (GMM) estimators introducing financial freedom and property right as additional instrumental variables.

The overall results indicate that H-statistic is positively related to financial stability and capitalisation, and negatively related to credit risk. Also, market power is negatively related to financial stability and capitalisation and positively related to credit risk. These results demonstrate that increases in competition and decreases in market power influence banks to hold more capital and take less credit risk enhance their financial stability. Such evidence strongly supports the competition-stability view of Boyd and Nicolo[5] for the commercial banks in ASEAN-5. The results also clarify a non-linear, inverted U-shaped relationship between competition and financial stability in the region, supporting the neutral view of Martinez-Miera and Repullo[6]. The results also indicate that the traditional measure of competition through concentration ratio is insufficient to explain the stabilising effects of competition in the ASEAN-5 market. The results further suggest that the impact of AFC on the competition-stability nexus was more severe than the recent GFC when ASEAN banks lost both their market power and capitalisation followed by excessive risk-taking.

This paper is organised into five sections. Section 2 presents a detailed review of the relevant literature explaining the nexus between competition and stability/fragility. Section 3 deals with the alternative estimation approaches of competition and financial stability and the model used to investigate the relationship. This section also describes the data sources. The results and their interpretation are reported in Section 4. Finally, Section 5 covers concluding remarks and policy recommendations.

## Literature review

A major concern of banking regulators and policy makers is to formulate policies that promote financial stability in the banking sector. Instability in the banking sector may contaminate the entire economy by sinking credit facility and distorting the interbank loan market and payment system. In investigating the cause of banking sector instability, Keeley [4] initiated an academic debate that theoretically and empirically showed that deregulation of the U.S. banking market during the 1970s and 1980s increases competition and renders banks fragile institutions. The debate is ongoing with conflicting theoretical forecasting and mixed empirical results.

The traditional view or competition-fragility view, also known as franchise value hypothesis, assumes that more competition leads banks to be more fragile [4, 11]. This hypothesis explains high competition in the financial market erodes market power, lowers the profit margin and capital buffer, and results in reduced franchise value that encourages the banks to adopt risk-taking strategies to increase returns. Advocates of this view consider that large banks dominate less competitive markets which are better able to benefit from economies of scale and scope, and better able to diversify their portfolios compared to smaller banks [12]. Moreover, a small number of large banks is also easy to monitor and supervise in a less competitive market [13]. Allen and Gale [13] further argue that banks earn less information rents from the relationship with borrowers in competitive markets. This provides banks less incentive to monitor the borrowers prudently which may give rise to moral hazard and adverse selection problem[14]. Others argue that contagion effect is more prominent in competitive markets as all banks are price takers and a solvent bank may not be interested to support liquidity to the troubled banks [15].

Conversely, the modern view or competition-stability view implies that high competition promotes the financial stability of financial institutions. Boyd and Nicolo [5] argue that banks with high market power enjoy lower competition in the loan market which encourages them to set high interest rates for borrowers which in turn increases their (borrowers) risk-taking tendency and default risk. They further argue that the bank will face moral hazard and adverse selection problem and lose solvency as the risk is ultimately transferred from the borrowers to the banks. Acharya, Gromb [16] argue that large banks in concentrated markets receive subsidies from policy makers through ‘too-big-to-fail’ or ‘too-important-to-fail’ schemes which alter their risk-taking motives and induce them to take extra risk, thus intensifying their fragility. The recent credit crunch is evidence that large banks are difficult to supervise due to their complexity and high political connection [17]. Furthermore, large banks in a concentrated market influence others through the contagion effect. Therefore, failure of large banks in a concentrated market renders the whole system fragile.

On the other hand, Martinez-Miera and Repullo [6] argue that the competition-stability nexus is non-linear and inverted U-shaped. This is because, high market power in less competitive loan markets induces banks to set high interest rates for the borrowers which not only increases the banks’ risk of insolvency, but also increases the profitability of the bank due to interest effect[6]. Similarly, Berger, Klapper [18] argue that the competition-fragility view and competition-stability view are not opposite perditions, rather both are concurrently applicable if high risk-taking can be hedged with a high capital buffer.

In addition, business cycle theory suggests that during recession banks adopt conservative approaches in credit management, shrink loan extension, and focus on building a capital buffer [19]. Such action can help banks to reduce loan exposure and moral hazard and improve stability. However, Cook [20] indicates that a few banks suffered from a moral hazard problem during the 1997–98 AFC. Hence, the effect of competition on financial stability becomes doubtful during financial crisis, as crisis changes the risk-taking initiative of the banks. Under this situation, banks may adopt a risk-taking policy to get benefit from safety-net subsidies or risk averse policies to reduce moral hazard.

There also exists a large body of literature studying the connection among financial stability, banking market concentration, and competition across multiple economies. For example, Yeyati and Micco [21] sampling of eight Latin American countries finds competition to be positively related to bank risk, but no relationship between market concentration and bank risk is found thus supporting the competition-fragility view.

Another cross-country study by Schaeck and Cihak [22] studies the link between banking system soundness and banking market competition for a sample of more than 3500 banks in ten European countries and around 9000 banks from the United States over the period of 10 years from 1995 to 2005. Schaeck and Cihak [22] find that boon indicator as a measure of market competition causes bank stability to increase by promoting banking efficiency and that financial stability benefits the more concentrated markets. Another study by Schaeck, Cihak [23] which takes a sample from 31 countries in financial crisis in 45 countries between 1980 and 2005, finds that chances of and time to crisis are reduced under competition after controlling for banking market concentration which shows a negative relationship with financial fragility.

Another cross-country study by Berger, Klapper [18] which uses more than 8000 banks as a sample from 23 countries suggests that banks with higher market power have less overall risk. Berger, Klapper [18] use the Lerner index as a measure of market power and Z-score as a measure of overall bank risk. Although their results favour the competition-fragility hypothesis, they also find that banks with market power have riskier loan portfolios. Another finding of Berger, Klapper [18] is that banks hold more equity to protect their charter value from risks arising out of loans.

Anginer, Demirguc-Kunt [24] uses the Lerner index and contingent claim pricing model of Merton [25] to study the link between banking market competition and banks’ default risk. Their analysis is based on a sample of 1850 banks from 63 countries world over. They find banking competition to be positively related to financial stability. Their results remain unchanged when they use market concentration as a proxy for market competition. Another study by Liu, Molyneux [26] investigates the link between bank competition and risk-taking in South East Asian countries. They use bank level risk indicators such as loan loss provisions, loan loss reserves, volatility of earnings and natural log of Z-score, and Panzar-Rosse H-statistic as a competition measure. They find evidence that competition does not lead to financial fragility.

A recent study by Fu, Lin [9] explores the relationship between bank competition and financial stability using bank-level data from 14 Asia-Pacific countries over the 2003–2010 period. This study considers Lerner index and large three banks’ concentration ratio as measure of competition, and contingent claim pricing model of Merton [25] along with Z-score as measure of banks’ risk-taking. Their study shows that the Lerner index is negatively related to risk-taking, while concentration is positively related to financial fragility of banks.

On average, cross-country studies provide mixed results regarding competition-stability nexus. However, there is evidence that both market concentration and market competition can coexist and that these impacts financial stability through different channels.

## Methodology

This study investigates the effect of bank competition on financial stability in ASEAN-5 commercial banks. It further investigates the non-linearity between competition and financial stability, following the works of Martinez-Miera and Repullo [6], Fu, Lin [9] and Kasman and Kasman [27]. Thus, we use the following general regression model, allowing for the aforementioned theoretical consideration:

$$\begin{array}{l} Stability_{ijt}=\alpha_{0}+\alpha_{1}Stability_{ijt-1}+\alpha_{2}Competition_{ijt}+\alpha_{3}{\left(Competition_{ijt}\right)}^{2}+\beta \;Bank\;Control_{ijt}\\ +\vartheta \;Regulatory\;Control_{jt}+\phi \;Crisis\;Dummy+\theta \;Macro\;Control_{jt}+\gamma {\left(year\right)}_{t}+\lambda_{i}+\varepsilon_{ijt}\;\forall_{ijt} \end{array}$$ (1)

In Eq 1, i = 1-----N, j = 1------J and t = 1-------T, N refers the number of individual banks; J refers the number of countries; T refers to time; and α, β, ϑ, ∅, θ and *γ* are estimated parameters. *Stability*_*ijt*_ denotes financial stability for bank *i* in country *j* at time *t* which is measured with Z-score, Equity ratio and NPL ratio *Competition*_*ijt*_ denotes level of competition or market power for bank *i* in country *j* at time *t* which is measured with both non-structural measure of competition, Panzar-Rosse H-statistic and Lerner index, and structural measure of competition, HHI. *Bank control*_*ijt*_ indicates bank characteristics for bank *i* in country *j* at time *t*. Bank characteristics include bank size, assets composition, operational efficiency and bank ownership. *Regulatory Control*_*jt*_ and *Macro Control*_*jt*_ are regulatory and macroeconomic control variables for country *j* at time *t*, where bank regulatory control variables include deposit insurance and activity restrictions, and macroeconomic control variables include annual real GDP growth rate and inflation rate. The effect of both 1997–98 AFC and 2008–2009 GFC on financial stability of ASEAN-5 also are investigated by incorporating crisis dummy. Here, two crisis dummies are included, where one for capturing 1997–98 AFC which takes 1 if the year is 1997 and 1998, otherwise zero; and another one for capturing 2008–2009 GFC which takes 1 if the year is 2008 and 2009, otherwise zero.

As suggested by Lee, Hsieh [28] to consider persistence of financial stability using a dynamic panel model, we included lagged dependent variable, *Stablity*_*ijt*−1_, in the model, where it’s coefficient *α*_1_ measures the persistence of financial stability of banks. A positive and significant value of *α*_1_ implies that financial soundness of one year is to be carried forward to the following year, implying banks’ persistent risk-taking behaviour. A year dummy is included to capture the year effect due to changes in the business cycle and technological progression. *λ*_*i*_ represents unobserved individual effects, and *ε*_*ijt*_ is the error term.

In the above Eq 1, the value of *α*_2_ and *α*_3_ are examined, such as, positive and significant value of both *α*_2_ and *α*_3_ for Z-score and Equity ratio while opposite for NPL ratio as dependent variable provide evidence to support competition-stability paradigm. This paradigm hypothesised that more competition or less market power induces banks to take less risk and to be more financially stable [5]. Conversely, negative and significant value of both *α*_2_ and *α*_3_ provide evidence to support competition-fragility paradigm. This paradigm hypothesised that more market power or less competition induces banks to take less risk and to be more financially stable [4]. Moreover, a different sign of *α*_3_ from *α*_2_ provides an evidence of non-linear or inverted U-shaped relationship between competition and financial stability as proposed by Martinez-Miera and Repullo [6].

### Measures of financial stability

This paper uses the Z-score as the primary measure of financial stability, following the works of Laeven and Levine [29], Soedarmono, Machrouh [30] and Schaeck and Cihák [31]. The theoretical underpinning of the Z-score is based on the work of Roy [32], which measures a bank’s distance from insolvency, where insolvency is a condition in which loss exceeds equity, such as (-π > E), where π stands for profit and E stands for equity. The probability of insolvency can be represented as probability (E/A < -ROA), where E/A is the equity asset ratio and ROA is the return on assets. The inverse of the probability of insolvency is (ROA + E/A)/δ(ROA), where δ(ROA) is the standard deviation of ROA. Thus, the Z-score is defined as the inverse of the probability of insolvency and indicates an individual bank’s soundness. The Z-score is calculated in the following manner:

$$Z_{ijt}=\frac{ROA_{ijt}+E_{ijt}/TA_{ijt}}{\delta \;ROA_{ijt}}$$ (2)

Where, *Z*_*ijt*_ is a measure of financial stability of *i* bank, in *j* country, at *t* time. *ROA*_*ijt*_ stands for the return on assets of *i* bank, in *j* country, at *t* time; *E*_*ijt*_/*TA*_*ijt*_ is a ratio of equity to total assets of *i* bank, in *j* country, at *t* time; *δ ROA*_*ijt*_ is a standard deviation of *ROA*_*ijt*_ Following the work of Soedarmono, Machrouh [30], we calculate *δ ROA*_*ijt*_ on the basis of the observation of *δ ROA*_*ijt*_ from time *t* to *t-2* (a three-year rolling window period, instead of the full sample period) to calculate the standard deviation of ROA. A higher Z-score value indicates the low probability of a bank’s financial distress, and its higher stability or financial soundness. The value of Z-score increases with an increasing level of profitability and capitalisation, and falls with an increase in the earnings volatility. We consider the natural logarithm of Z-score in order to normalize its value following the works of Laeven and Levine [29], Soedarmono, Machrouh [30].

We also use two alternative measures of financial distress and risk-taking measurements, such as NPL ratio and equity ratio. These alternative risk measures are used to understand whether the change in financial soundness occurs due to a change in credit risk, or an increase in capitalisation. The NPL ratio measures a bank’s loan portfolio risk or credit risk position. Previous studies use the NPL ratio include Jiménez, Lopez [33], Agoraki, Delis [10], and Amidu and Wolfe [34]. This is because credit risk is the primary banking risk, and its increase results in non-performing loans in the bank’s loan portfolio. A higher ratio indicates a bank’s higher tendency to keep a riskier loan portfolio, which undermines the bank’s financial soundness. Berger, Klapper [18] proposed using the equity ratio as an indicator of risk-taking, arguing that high capitalisation may offset the negative consequence of high credit risk on financial institutions’ overall risk. Further, the competition-fragility hypothesis of Keeley [4] argues that high market power allows banks to enjoy monopoly rents which stimulate them to take less risk, as monopoly rents are used to build capital buffer. Subsequently, a number of studies also use equity ratio as a risk-taking indicator, such as in the work of Laeven and Levine [29], Soedarmono, Machrouh [30] and Fang, Hasan [35]. The higher capitalisation ratio may enhance financial stability by offsetting banks’ risk-taking initiative.

### Measures of competition

H-statistic, based on the methodology of Panzar and Rosse [36], is used as a competition measurement. The methodology of Moch [37] is particularly followed in this study in determining H-statistic for each calendar year separately for each ASEAN-5 country, using the following reduced-form revenue regression model:

$$lnP_{it}=\alpha +\beta_{1}lnW1_{it}+\beta_{2}lnW2_{it}+\beta_{3}lnW3_{it}+\gamma_{1}lnX1_{it}+\gamma_{2}lnX2_{it}+\gamma_{3}lnX3_{it}+\varepsilon_{it}$$ (3)

The subscript *ln* indicates the natural logarithm, *i* indicates bank, *t* indicates time, *P*_*it*_ is the measure of output price of the loan, which is calculated by dividing interest income to total assets following an intermediation approach, and *ε*_*it*_ is the error term. *W*1_*it*_ is the ratio of interest expenses to total assets as a ratio of the price of borrowed funds, *W*2_*it*_ is the ratio of personnel expenses to total assets as a measure of the price of labor, and *W*3_*it*_ is the ratio of administrative and other operating expenses to total assets as a measure of the price of fixed capital. Three bank-specific control variables, *X*1_*it*_, *X*2_*it*_, and *X*3_*it*_, were added as the ratio of customer loan to total assets, ratio of equity to total assets, and total assets in millions of USD, respectively, as these are expected to influence the bank’s revenue function.

H-statistic is calculated as a sum of the elasticities of bank’s total revenue, with respect to the above input prices, calculated as *H* = *β*_1_ + *β*_2_ + *β*_3_. The H-statistic may take a value from -∞ to +1. A larger H-statistic indicates the change in input prices’ greater influence on total revenue and more market competition. The value of H-statistic in perfect competition is equal to one, or that the proportion of increase in input prices and total revenue is the same. This is because the firm exits the market if it does not cover input prices. H-statistic under a monopoly take either a zero or negative value, which means that an increase in input prices reduces the bank’s total revenue. Under monopolistic competition, it takes a value between zero and one.

The following regression specification is used to test whether the H-statistic satisfies the long run equilibrium condition, as the existence of a disequilibrium condition may invalidate the value of the H-statistic [37, 38]:

$$ln\left(1+ROA_{it}\right)=\alpha +\beta_{1}lnW1_{it}+\beta_{2}lnW2_{it}+\beta_{3}lnW3_{it}+\gamma_{1}lnX1_{it}+\gamma_{2}lnX2_{it}+\gamma_{3}lnX3_{it}+\varepsilon_{it}$$ (4)

Where, *ROA*_*it*_ is return on assets for bank *i* at time *t*. In a long run equilibrium condition, *β*_1_ + *β*_2_ + *β*_3_ = 0, indicating that input prices do not affect the bank’s return on assets.

This study also uses the Lerner index developed by Lerner [39] to measure competition, which is also an extensively used measure of banking competition in recent literature, such as Jiménez, España [40], Berger, Klapper [18], Deniz, Asli [41], Nguyen, Skully [42], Amidu and Wolfe [34], Liu and Wilson [43], Soedarmono, Machrouh [30] and Fu, Lin [9]. The Lerner index measures the mark-up of price over the marginal cost; the deviation of price from marginal cost is considered a market power. The value of the Lerner index ranges from 0 to 1. A higher Lerner index value indicates banks’ higher market power, to set the product price over marginal cost and low competition. Product price and marginal cost are both equal in a perfectly competitive market; namely, the Lerner index = 0; in a pure monopoly market, the Lerner index = 1. The non-optimal behaviour of the market participant in setting product price is represented by the Lerner Index < 0, where the bank loan is priced below the marginal cost. The Lerner index is measured in the following manner:

$$Lerner_{it}=\frac{P_{TA_{it}}-MC_{TA_{it}}}{P_{TA_{it}}}$$ (5)

Where, ${P_{TA}}_{it}$ is the price of total assets, indicating the ratio of total revenue to total assets for bank *i* at time *t*. Total revenue is sum of interest income, non-interest operating income and other operating income following the work of Anginer, Demirguc-Kunt [24]. ${MC}_{{TA}_{it}}$ is the marginal cost of the total assets of bank *i* at time *t*. The following translog cost function is estimated for each ASEAN-5 country, using the methodology of Demirguc-Kunt and Pería [3] and Anginer, Demirguc-Kunt [24], to estimate ${MC}_{{TA}_{it}}$:

$$\begin{array}{l} lnCost_{it}=\\ \alpha +\beta_{1}ln\left(Q_{it}\right)+\beta_{2}ln{\left(Q_{it}\right)}^{2}+\beta_{3}ln\left(W1_{it}\right)+\beta_{4}ln\left(W2_{it}\right)+\beta_{5}ln\left(W3_{it}\right)+\beta_{6}ln\left(Q_{it}\right)ln\left(W1_{it}\right)+\\ \beta_{7}ln\left(Q_{it}\right)ln\left(W2_{it}\right)+\beta_{8}ln\left(Q_{it}\right)ln\left(W3_{it}\right)+\beta_{9}ln{\left(W1_{it}\right)}^{2}+\beta_{10}ln{\left(W2_{it}\right)}^{2}+\beta_{11}ln{\left(W3_{it}\right)}^{2}+\\ \beta_{12}ln\left(W1_{it}\right)ln\left(W2_{it}\right)+\beta_{13}ln\left(W2_{it}\right)ln\left(W3_{it}\right)+\beta_{14}ln\left(W1_{it}\right)ln\left(W3_{it}\right)+\theta \;Year\;Dummy+\varepsilon_{it} \end{array}$$ (6)

The subscript *ln* in Eq 6 indicates the natural logarithm, *i* indicates banks, and *t* indicates year. Cost is the sum of interest expenses, non-interest operating expense, personnel expenses, other administrative expenses, and other operating expenses, expressed in millions of USD [24]. *Q*_*it*_ is total assets in millions of USD, representing output quality. Three input prices are then used to capture the price of borrowed funds (*W*1_*it*_), the price of labor (*W*2_*it*_), and fixed capital (*W*3_*it*_). *W*1_*it*_ is the ratio of interest expenses to total assets, *W*2_*it*_ is the ratio of personnel expenses to total assets, and *W*3_*it*_ is the ratio of administrative and other operating expenses to total assets. The cost function is estimated separately for each country to account for potential technological differences among the countries, following the work of Berger, Klapper [18]. A year dummy is included to handle technological progress and changes to the business cycle’s condition. Additionally, the following five restrictions are imposed to ensure homogeneity of degree one in the input prices:

$$\beta_{3}+\beta_{4}+\beta_{5}=1;\;\beta_{6}+\beta_{7}+\beta_{8}=0;\;\beta_{9}+\beta_{12}+\beta_{13}=0;\;\beta_{10}+\beta_{12}+\beta_{14}=0;\;\beta_{11}+\beta_{13}+\beta_{14}=0$$

The coefficient of Eq 6 is used to estimate the marginal cost for bank *i* at time *t*, using the following equation:

$$MC_{it}=\frac{\partial C_{it}}{\partial Q_{it}}=\frac{C_{it}}{Q_{it}}\left[\beta_{1}+2\beta_{2}lnQ_{it}+\beta_{6}lnW1_{it}+\beta_{7}lnW2_{it}+\beta_{8}lnW3_{it}\right]$$ (7)

Recent literature such as Kasman and Kasman [27], Anginer, Demirguc-Kunt [24], Jiménez, Lopez [44] and Xu, Rixtel [45] use concentration measures as a proxy of competition. This is because, concentration and competition could coexist and indicate a banking system’s stability and fragility. Therefore, in addition to the above-mentioned new empirical industrial organisation approaches of competition, Lerner index and H-statistic, we also use traditional measure of concentration HHI in order to investigate the effect of concentration on stability in ASEAN-5. Where, HHI is calculated as the sum of the squares of the market share of each bank in the loan market following the work of Berger, Klapper [18].

### Control variable

A number of bank-specific, regulatory, and macroeconomic variables are controlled in examining the relationship between competition and financial stability. Bank-specific control variables are bank size, assets composition, operational efficiency, and foreign ownership. Where, bank size is the natural logarithm of total assets. This is controlled as Liu, Molyneux [26] argue that large banks may take more risk due to higher market power; thus, size significantly effects a bank’s financial stability. The assets composition, which is the ratio of net loan to total assets, is also controlled as this measures banks’ lending behaviour. Kasman and Kasman [27] find that a high lending rate increases banks’ credit risk and overall risk. The cost to income ratio is also controlled to account for the banks’ operational efficiency, as Schaeck and Cihák [31] find that efficiency is the channel through which competition affects financial stability. Further, Boyd and Nicolo [5] and Fiordelisi and Mare [46] discovered evidence that less efficient banks expose their operations to a higher risk to improve performance and generate higher returns. A foreign bank dummy is also controlled, following the work of Berger, Klapper [18], as foreign banks may have higher efficiency and capitalisation, and the improved ability to manage banking risk.

Dummies for both the AFC and GFC are also controlled for the relationship between competition and stability, which may be altered due to financial crisis. This is because crisis causes the banking market to endure restructuring processes, which alters banks’ market power and risk-taking appetites. Regulatory variables are also controlled in examining the relationship between competition and stability, following the work of Beck, De Jonghe [47] and Fu, Lin [9], as certain types of regulation may affect banks’ market power and change its risk-taking behaviour. Bank regulation makes the nexus between competition and financial stability robust, and also offer additional information regarding the nexus [47].

Deposit insurance is captured with a dummy variable, which takes a value of 1 for the country with an explicit deposit insurance scheme, and 0 for otherwise. Deposit insurance is expected to enhance the banking system’s financial stability by preventing the bank from risk-taking in a competitive market. However, this depends on prudent supervision of the insured institution’s risk-taking and capital positions. Otherwise, insurers would incur loss exposure, which weakens financial stability. Activity restrictions were also controlled, which may affect the competition-stability nexus by eroding market power to involve certain types of activities. An activity restrictions index was constructed to determine whether banks are (1) unrestricted, (2) permitted, (3) restricted, or (4) prohibited in a country for its involvement in insurance, securities, real estates, and ownership of non-financial firms. The index ranges from 4 to 16, and the higher index value indicates higher restrictions on banking activities. A real GDP growth rate is considered, following the work of Agoraki, Delis [10] as this implies fluctuations of economic activities, or a movement in the business cycle, which is likely to affect the country’s financial institutions’ performance. Inflation, or the consumer price index’s annual growth rate, is also controlled following the work of Lee and Hsieh [48] as a proxy of macroeconomic instability due to its inverse effect on the real economy. S1 Table provides a summary of the variables used in the analysis incorporating their definition, sources and expected sign.

### Estimation method and data

In the investigation process, the study opts to use dynamic panel model because it captures dynamic nature of financial stability and potential endogeneity problem between financial stability and bank competition. Also, it offers better outcomes compared to a static model which uses random effect and/or fixed effect models. Where, random and/or fixed effect model provides serious econometric bias and inconsistent results due to the presence of correlation between error term and lagged dependent variables [49]. To deal with such correlation between error term and lagged dependent variable, instrumental variable techniques are used. We choose to apply here Generalized Method of Moments (GMM) estimators proposed by Arellano and Bond [50] to better estimate the dynamic relationship between competition and financial stability. More particularly, we apply two-step system GMM of Arellano and Bover [51] and Blundell and Bond [52] to attain perfect estimators. The two-step system GMM is ideal for such conditions where the number of period (T) is small and cross sections (N) is large; dependent variable is persistent (dynamic); explanatory variables are not exogenous (they may correlate with error term), there are heteroscedasticity, time-invariant individual fixed effect and autocorrelation within individuals, which are more common in bank level data.

Arellano and Bond [50] originated the standard GMM estimator, also known as first-differenced GMM, where all variables are transformed by differencing and introduced instrument variables from the lagged levels of the regressors. However, the lagged levels of the regressors could be a poor instrument if there is a serial correlation in the errors. In this case, first difference GMM might result in imprecise or even biased estimators. To overcome these shortcomings, Arellano and Bover [51] and Blundell and Bond [52] developed the system GMM which comprises two simultaneous equations, whereby, one equation is in lagged difference of the dependent variable as instruments for equation in levels, and other is in lagged levels of dependent variables as instruments for equation in first difference. Blundell and Bond [52] demonstrate that the System GMM has smaller variances and is more efficient, thereby improving the precision in the estimator. In investigating competition-financial stability relationship, we consider financial freedom and property right as external instruments for controlling potential endogeneity problem of competition with financial stability based on economic arguments following the work of Berger, Klapper [18] and Fu, Lin [9]. Where, financial freedom measures the efficiency as well as the freedom of the banking system from government intervention and control in the forms of banking regulations, credit allocation, deposit accumulation, types of financial services offering, dealing with foreign currencies, and foreign ownership in the banking system. Financial freedom is expected to change the market power of the banking system, and thus influences financial stability. Likewise, property right determines the level to which private property right is protected by the laws and its enforceability by the government. The property right is also expected to affect both competition and financial stability of a banking system, because it encourages banks to innovate new products and services which help them capture the market share, and drives out the less efficient banks from the market.

Before running two-step system GMM, the presence of autocorrelation, heteroscedasticity, and endogeneity of the data set is tested applying Wooldridge test, Breusch-Pagan/Cook-Weisberg test, and the Wu-Hausman test, respectively. After running the two-step system GMM some post-diagnostic test were also performed, such as AR(1) and AR(2) to test presence of autocorrelation at first and second difference respectively, first stage F-test using 2SLS estimator to test relevance, and Hanen’s J-test to test the validity of instruments of endogenous variables, such as competition measures. Wald test is also used to ensure the goodness of fit for all our regression models.

Bank-level data are retrieved from the BankScope database, developed by Bureau Van Dijk, to construct a sample of an annual, unbalanced panel from 1990 to 2014, which covers both the 1997–1998 AFC and the 2008–2009 GFC. Banks are eliminated from the initial sample with less than three consecutive years’ observations, as well as banks with high missing values in income statement variables used to calculate the Lerner index and H-statistic, following the work of Chan, Koh [53]. To avoid survivorship bias, we have included as many banks as possible considering also those that are not active during last 25 years. Thus, the result is unbalanced panel data from 2527 observations at 180 commercial banks in ASEAN-5 nations. Following the works of Liu, Molyneux [26], Nguyen, Skully [42], and Fu, Lin [9] who study Asian banks, the focus is only on commercial banks, as commercial banks account for more than 82% of financial assets in ASEAN-5 countries[8]. Moreover, commercial banks are expected to be more competitive than other types of banks because of additional exposure to competition from capital markets and foreign competitors [54]. Additionally, these banks tend to have more freedom in choosing their business mix and face similar restrictions across countries. Furthermore, we have excluded other types of banks (such as investment banks, saving banks and cooperative banks) and non-bank financial institution (such as insurance, leasing, etc.) to confirm comparability of regulatory restrictions. This is because regulatory restrictions on commercial banks are different from other entities. All income statement data and ratios, such as a non-performing loan to a gross loan, equity to total assets, net loan to total assets, and cost to income ratio, were winsorized at the first and ninety-ninth percentiles to eliminate outliers and reduce the estimation error.

The dependent variables in the analysis are the Z-score, NPL ratio, and equity ratio. Measures of competition were used as main explanatory variables which include both non-structural measures, such as H-statistic and Lerner Index and structural measure through a concentration ratio, such as HHI. Bank level variables were then controlled, such as bank size, asset composition, operational efficiency, and foreign ownership, which are captured through a natural logarithm of total assets, the ratio of net loan to total assets, the ratio of cost to income, and a foreign ownership dummy, respectively. The BankScope database and individual bank websites were searched to collect bank ownership data. A bank is considered to be a foreign bank if the market share of its foreign owners exceeds 50%. Regulatory variables were also controlled, such as activity restrictions, deposit insurance, and macroeconomic variables, such as the annual real GDP growth rate, and the inflation rate based on the CPI. Banking regulation data is collected from the World Bank regulation and supervision database, developed by Barth, Caprio [55] and updated by Barth, Caprio [56, 57]. As data is only available at certain points in time, information was used from the first, second, and third surveys to observe 1998–2000, 2001–2003, and 2003–2008, respectively. The data relating to macroeconomic conditions is collected from the World Bank Development Indicator (WB-DI).

## Empirical analysis

### Descriptive statistics and correlation structure

Table 1 reports descriptive statistics of the bank-level variables used for each country in the region, and also for ASEAN-5 nations. Table 1 depicts that the banking stability of Singapore and the Philippines is higher in the region, with an average Z-score that is higher than that of the ASEAN-5. Conversely, the least financially stable banks come from Thailand and Indonesia. This table also illustrates that banks from Thailand and Indonesia face more loan portfolio risk and less capitalisation. This table further reports that Malaysian and Singaporean banks enjoy the more market power, and face less competition in the region, which is observed from the higher value of Lerner index, and lower value of H-statistic. Loan concentration based on HHI, on the other hand, is higher for Singapore, followed by Malaysia. Additionally, the primary banks that originated from Singapore and Thailand followed by Malaysia, and regionally, smaller banks come from Indonesia and the Philippines, having a higher average of total assets. The level of intermediation, captured with the ratio of net loan to total assets, is at its maximum in Thailand, followed by Indonesia and Singapore, and less in the Philippines and Malaysia. However, more efficiency in banking operations, based on the cost to income ratio, comes from Malaysian banks, followed by Singapore and Indonesia. In term of banking regulations, banks from Malaysia and the Philippines face more restrictions to involve insurance and other activities, and banks from Indonesia, followed by Singapore, face fewer restrictions. All banks, except banks from Thailand, enjoy an explicit deposit insurance scheme from the insurer in their banking operations.

**Table 1:** ASEAN-5 and country wise descriptive statistic of the variables.

Variables	ASEAN-5				Indonesia	Malaysia	Philippines	Singapore	Thailand
	Mean(S.D.)	Maximum	Median	Minimum	Mean(S.D.)	Mean(S.D.)	Mean(S.D.)	Mean(S.D.)	Mean(S.D.)
Input and output variables									
Price of fund	.091(.28)	.761	.043	.001	.132(.38)	.04(.03)	.097(0.28)	.041(.03)	.048(0.05)
Price of labor	.011(.042)	.166	.009	0	.012(.01)	.006(0.00)	.016(0.02)	.018(.14)	.010(0.01)
Price of capital	.016(.02)	.209	.012	-.001	.02(.02)	.006(0.00)	.02 (.02)	.01(.06)	.015(0.01)
Price of loan	.209(.22)	.816	.172	-.858	.28(.29)	.167(.094)	.191 (.11)	.117(.19)	.132(.05)
Price of output	.080(.05)	.502	.072	0	.11(.05)	.049(.02)	.075 (.04)	.036(.02)	.063(.03)
Total cost	386.97 (675.74)	5301.6	131.713	-3	271.67 (554.66)	339.753 (518.75)	232.954 (283.81)	967.103 (1316.68)	687.022 (786.52)
Total assets	7518.51 (19471.47)	288590.10	177	2.067	2930.25 (6747.35)	7367.501 (12429.25)	3089.693 (4344.02)	33996.013 (55338.21)	13046.833 (14262.82)
Dependent Variables: Financial Stability Measures									
Z-score	76.63(163.72)	354.12	39.464	-8.822	65.18(147.16)	71.57(119.19)	101.74(232.61)	113.62(139.30)	63.34(155.60)
NPL ratio	8.579(11.86)	62.550	4.576	0.03	8.577(13.32)	5.888(7.90)	11.277(13.63)	3.437(3.12)	11.206(10.28)
Equity ratio	12.564(12.58)	56.147	10.569	.09	11.794(14.85)	10.851(6.99)	14.674(8.41)	18.774(17.59)	10.752(10.26)
Endogenous Variables: Competition Measures									
Lerner index	.116(.33)	.648	.241	-.775	.114(.32)	.314(.22)	-.069(.38)	.215(.35)	.064(.28)
H-Statistic	.550(.28)	1.497	.567	-.475	.596(.18)	.500(.23)	.478(.32)	.321(.35)	.698(.37)
HHI in loan	.130 (.05)	.454	.119	.076	.098(.03)	.134(.04)	.125(.01)	.304(.01)	.129(.03)
Control Variable: Bank level									
Assets composition	55.248(19.82)	99.700	59.150	0	56.01(18.89)	51.057(21.03)	46.795(15.60)	52.549(23.48)	70.337(14.18)
Bank size	7.472(1.92)	12.669	7.475	.617	6.636(1.77)	8.152 (1.51)	7.200(1.57)	8.606(2.36)	8.766(1.57)
Foreign ownership	.340(.47)	1	0	0	.345(.47)	.339(.47)	.320(.46)	.388(.48)	.327(.47)
Operational efficiency	59.901(48.79)	873.580	52.227	.662	60.81(51.34)	40.473(25.53)	71.625(47.59)	51.125(24.83)	70.899(64.03)
Control Variable: Regulatory and macro-economic									
Activity restrictions	11.050 (2.19)	15	11	8	8.918(.99)	13.327(.47)	13(0)	10(0)	12.501(1.83)
Deposit insurance	.668(.47)	1	1	0	1(0)	.5192(.50)	.648(.48)	.537(.49)	0(0)
Inflation	6.575(8.51)	58.387	5.047	-.846	10.739(11.65)	2.565(1.24)	5.539(2.80)	2.031(1.73)	3.300(2.07)
Real GDP growth	4.677(3.95)	15.240	5.317	-13.13	4.429(4.32)	5.350(3.82)	4.459(2.11)	6.196(4.25)	4.023(4.34)
Observations	2527				1050	441	466	201	367

Note: This table provides descriptive statistics of the variables for ASEAN-5 combinedly, and isolated manner for each country including Indonesia, Malaysia, the Philippines, Singapore and Thailand. Price of fund is the ratio of interest expenses to total assets, price of labor is the ratio of personnel expenses to total assets, price of capital is the ratio of administrative and other operating expenses to total assets, price of loan is ratio of total revenue to total assets, price of output is ratio of interest income to total assets. The input and output variables are used to calculate Lerner index and H-statistic. Other variables and data sources are defined in S1 Table. Value in the parenthesis indicates standard deviation.

**Fig 1:**
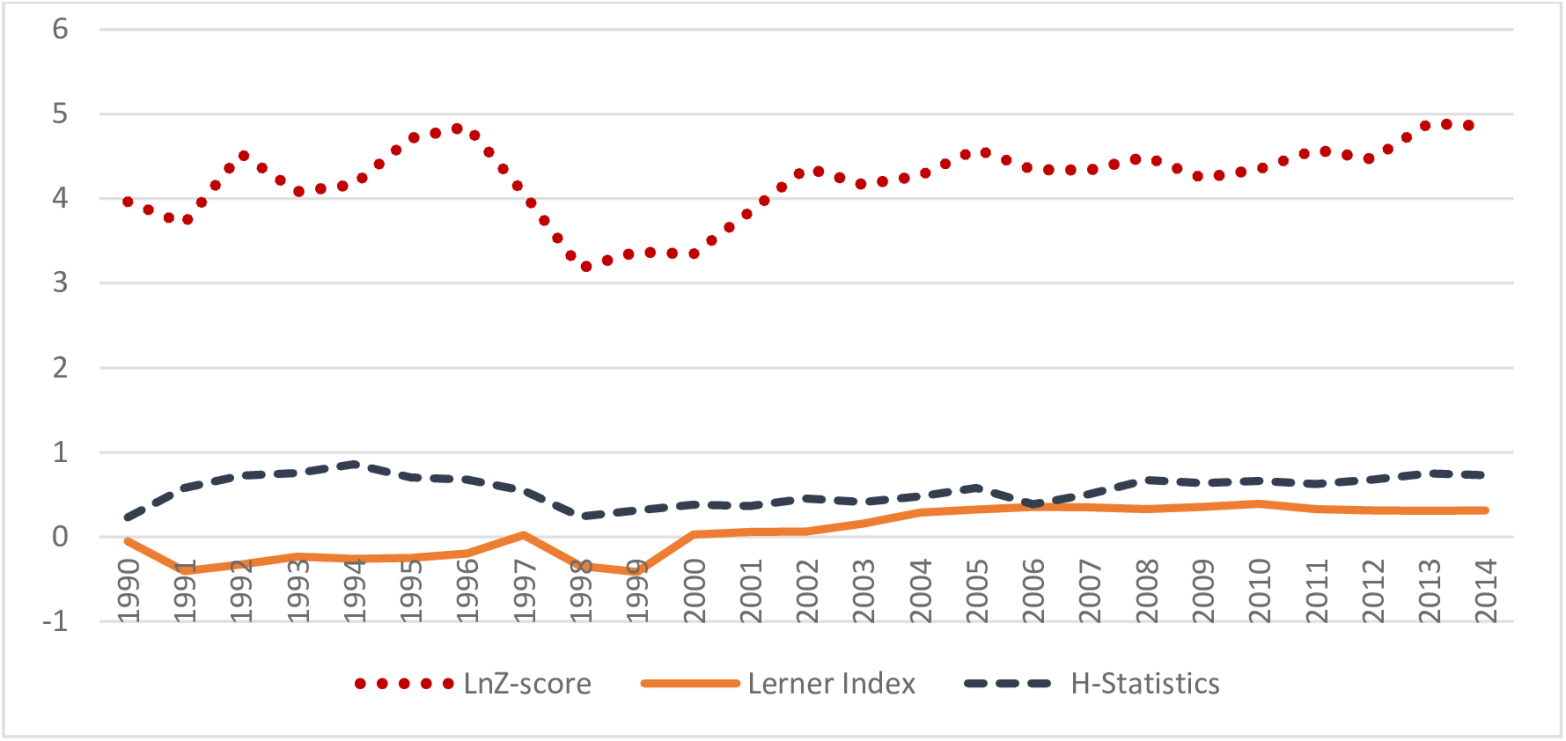
Co-movement of lnZ-score, H-statistic and Lerner Index in ASEAN- 5 during 1990–2014 period. Note: *Fig* 1 presents co-movement lnZ-score, H-statistic and Lerner index in ASEAN-5 from 1990 to 2014. ASEAN-5 includes Indonesia, Malaysia, the Philippines, Singapore and Thailand. Definition of lnZ-score, H-statistic and Lerner index, and source of their data collection are presented in S1 Table.

**Fig 2:**
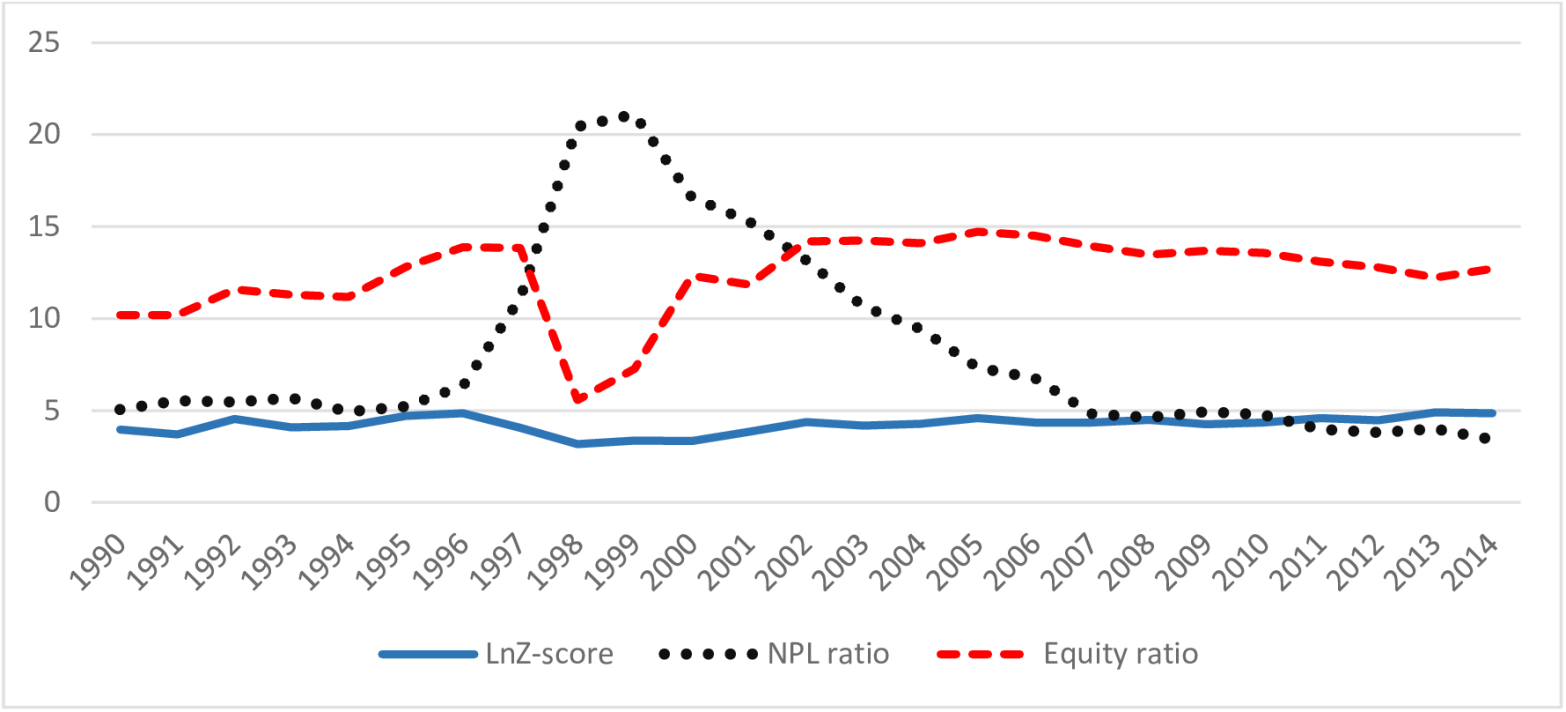
The relationship among lnZ-score, NPL ratio and Equity ratio in ASEAN-5 from 1990–2014 period. Note: Fig 2 presents the relationship among lnZ-score, NPL ratio and equity ratio in ASEAN-5 from 1990 to 2014. ASEAN-5 includes Indonesia, Malaysia, the Philippines, Singapore and Thailand. Definition of lnZ-score, NPL ratio and equity ratio, and source of their data collection are presented in S1 Table.

Table 2 presents H-statistic, the Lerner index, and HHI as competition measures, and the Z-score, NPL ratio and equity ratio as financial stability measures for ASEAN-5 nations on an annual basis from 1990–2014 period. Table 2 are presented through *Fig 1* and *Fig 2* for better understanding the relationship between competition and financial stability in this region.

**Table 2:** Yearly average of H-statistic, Lerner index, and HHI based on loan based on loan for ASEAN-5 during 1990–2014.

Year	H-statistic	Lerner	HHI(Loan)	Z-score	NPL ratio	Equity ratio	Observation
1990	.229	-.054	.218	52.687	5.055	10.190	22
1991	.577	-.405	.215	40.483	5.538	10.181	26
1992	.721	-.327	.201	92.551	5.451	11.571	40
1993	.754	-.237	.198	59.355	5.674	11.292	50
1994	.859	-.262	.159	64.266	4.942	11.172	69
1995	.704	-.251	.154	111.1425	5.221	12.805	78
1996	.676	-.200	.143	128.156	6.347	13.887	82
1997	.545	.020	.138	58.270	11.145	13.839	109
1998	.239	-.342	.141	23.930	20.423	5.564	96
1999	.314	-.419	.138	29.037	21.11	7.262	102
2000	.379	.027	.122	28.298	16.606	12.328	109
2001	.364	.058	.121	46.576	15.285	11.849	106
2002	.451	.062	.119	78.307	13.16	14.178	110
2003	.410	.157	.118	64.413	10.65	14.228	117
2004	.479	.284	.116	71.511	9.43	14.097	118
2005	.575	.324	.129	97.366	7.326	14.709	108
2006	.384	.351	.123	76.636	6.753	14.490	116
2007	.506	.349	.123	76.614	4.804	13.934	117
2008	.670	.325	.124	89.361	4.627	13.469	117
2009	.633	.351	.125	69.544	4.939	13.694	122
2010	.658	.390	.120	77.183	4.769	13.564	124
2011	.622	.329	.117	96.927	3.973	13.081	120
2012	.676	.310	.115	86.849	3.797	12.781	134
2013	.748	.304	.115	132.511	3.994	12.232	133
2014	.727	.309	.115	128.505	3.425	12.694	120

Note: This table reports yearly average value of H-statistic, Lerner index, HHI, Z-score, NPL ratio and equity ratio for ASEAN-5 from 1990 to 2014. ASEAN-5 includes Indonesia, Malaysia, the Philippines, Singapore and Thailand. The description of variables and sources of the data are provided in S1 Table.

***Fig*** 1 depicts ASEAN-5’s natural log of Z-score, H-statistic, and Lerner index for the same period, to better understand the nature of the competition-stability nexus. This figure demonstrates that the Z-score moves cyclically with H-statistic, but it moves with the Lerner Index counter cyclically. A sharp decline in the Z-score’s log during the AFC in 1997–1998; after this event, it increases, albeit with some fluctuations. The same trend was also observed in H-statistic, while reversing in Lerner index, indicating an overall banking risk sharply increased during the AFC due to a sharp decline in the level of competition. It is evident from the Lerner index that ASEAN-5 banks’ market power was negative until 1997, then runs negative again until 1999. The negative market power means higher marginal cost in comparison to loan price resulting from banks’ non-optimum behavior, as indicated by Soedarmono, Machrouh [30]. This is because, the period of ASEAN-5 banks’ negative market power is characterized by financial deregulation and the 1997–98 AFC. The non-optimum behavior of the ASEAN-5 banks in aforementioned period is also supported by Corsetti, Pesenti [58] who claim that financial liberalization in east Asian countries including ASEAN-5 in early 1990s increased bank lending and operational costs, and decreased bank profitability. These are resulted from structural distortion including weak regulation and lax supervision, less expertize in regulatory institutions, low capital adequacy ratio, absence of incentive compatible deposit insurance scheme and corrupt bank lending[58]. Katib and Mathews [59] reported that financial liberalization has resulted not only increase in the number of banks in Malaysia, but also higher operational costs and negative technological progress. Williams and Intarachote [60] claim that profit inefficiency has increased progressively in the banks from Thailand during 1990–1997 as a consequence of deregulation induced banking activity expansion. Karim [61] claims that cost inefficiency was divergent among the commercial banks in Indonesia, Malaysia, the Philippines and Thailand, and it also increased progressively in the preceding years of 1997–98 AFC from 1989 to 1996 period. Karim [61] further identified that state-owned banks suffered from high cost inefficiencies during 1989–1996; and Laeven [62] identified that during the period of financial liberalization and the 1997–98 AFC period the banking industry of Indonesia, Singapore and Thailand was largely dominated by state-owned banks.

On the other hand, the log of the Z-score during the 2008–2009 GFC, reveals that initially, the Z-score declined, then moves upward, consistent with the work of Fu, Lin [9]. This implies that the region was initially affected with the GFC, but dramatically recovered from that crisis. During the same period, it is observed a downward slope in H-statistic and an upward slope in Lerner index until 2009, implying that during the 2008–2009 GFC, ASEAN banks suffered from high-risk pressure due to decreased in competition and increased market power. This trend of market power featured by Lerner index during GFC found consistent with the work of Fu, Lin [9], who investigated the market power of the Asia-Pacific region using the Lerner index during 2003 to 2010.

Again, ***Fig 1*** demonstrates that the lnZ-score and H-statistic are found increasing, and Lerner index is found decreasing from 2011 when ASEAN central banks endorsed ABIF which allows qualified banks from ASEAN-5 to expand cross-border operations in other member states of ASEAN-5 with home field advantage. This implies that ABIF found enhancing financial stability by increasing the level of competition and eroding the market power of the banking system. ***Fig 2*** shows that the equity ratio moves cyclically and NPL ratio moves counter-cyclically with *ln*Z-score. This demonstrates that high equity ratio may enhance financial stability along with Z-score, while high NPL ratio may undermine it.

Finally, Table 3 illustrates the Pearson pair-wise correlation of independent non-dummy variables used in the models, as well as the level of significance. The table importantly demonstrates that regressors are not highly correlated between them albeit their coefficients are significant, because, their pair-wise correlation coefficients are less than 0.50. Thus, the regressors are free from multicollinearity problem.

**Table 3:** Pearson pair wise correlation matrix of independent variables used in the analysis.

	(1)	(2)	(3)	(4)	(5)	(6)	(7)	(8)	(9)
H-statistic (1)	1.000								
Lerner index (2)	.140***	1.000							
HHI (3)	-.061***	-.108***	1.000						
Assets composition (4)	.114	-.114***	-.011	1.000					
Bank size (5)	.089***	.204***	.203***	.157***	1.000				
Operational efficiency (6)	-.029	-.102***	-.072***	-.095***	-.147***	1.000			
Activity restrictions (7)	-.14***	-.211***	.204***	-.034*	.191***	-.008	1.000		
Inflation rate (8)	-.123***	-.379***	-.127***	-.095***	-.29***	-.012	-.216***	1.000	
GDP growth rate (9)	.286***	.270***	.102***	.067***	.128***	-.042***	-.031	-.494***	1.000

Note: This table provides Pearson pair-wise correlation of the variables of this study. The description of the variables and sources of data are provided in S1 Table.

*** and * indicating the coefficient are significant at 1 percent and 10 percent respectively

## Results and discussion

Tables 4 and 5 present two-step system GMM regression results of financial stability models specified in Eq 1. Here, we estimate six models in Table 4, where *ln*Z-score and equity ratio are used as dependent variable in models (1–3) and (4–6) respectively, and as measures of competition, such as models (1) and (4) use H-statistic, models (2) and (5) use Lerner index and models (3) and (6) use HHI. In addition, we estimate three models in Table 5, where NPL ratio is used as dependent variable and as competition measure model (1) uses H-statistic, model (2) uses Lerner index and model (3) uses HHI. The diagnostic tests for two-step system GMM are provided at the bottom of the Tables 4 and 5. The significant value of Wooldridge test, Breusch-Pagan/Cook-Weisberg test and Wu- Hausman test show there involves serial correlation, heteroscedasticity and endogeneity. In order to handle the serial correlation, heteroscedasticity and endogeneity issue, this study adopts the instrumental variable technique. As a first attempt, it runs first stage F-test using 2SLS regression in order to check instrumental validity, the significant value of first stage F statistics indicates that the instruments are weak and invalid in 2SLS regressions and fixed effect instrumental variable estimator are also likely to be biased in the way of OLS estimators. Therefore, the study uses two-step system GMM of Arellano and Bover [51] and Blundell and Bond [52], as Roodman [63] argued that the two-step system GMM improves precision of estimates controlling serial correlation, heteroscedasticity and endogeneity problems.

**Table 4:** The effect of competition measured by H-statistic, Lerner index and HHI on *ln*Z-score and equity ratio as measure of financial stability in ASEAN-5 from 1990–2014 period.

Dep. Variable	*ln*Z-score			Equity ratio		
Model	Model (1)	Model(2)	Model (3)	Model (4)	Model (5)	Model (6)
Lagged lnZ-score, equity ratio	.644(.028)***	.250(.040)***	.310(.034)***	.253(.051)***	.248(.055)***	.239(.052)***
H-statistic	.505(.242)**			2.149(1.882)		
H-statistic^2	-.381(.208)*			-.374 (1.395)		
Lerner		-1.221(.247)***			-2.054(1.111)*	
Lerner^2		-.3722 (.356)			-.201(1.811)	
HHI			-.388(6.241)			-21.473(45.482)
HHI^2			3.571(15.72)			90.329(129.261)
Inflection point	+0.66	-1.64	+0.05	2.87	-5.11	0.12
Relationship(sign)	+	-	-	+	-	-
Loan to Assets	.005(.004)	-.005(.007)	.010(.005)**	-.041(.020)**	-.114(.036)***	-.047(.028)*
Bank Size	.012(.034)	.322(.071)***	.088(.047)**	-.833(.235)***	-.446(.359)	-1.278(.354)***
Foreign Ownership	.049(.093)	.087(.109)	-.026(.086)	1.111(.846)	1.663(.935)*	1.016(.664)
Operational Efficiency	-.009(.002)***	-.024(.004)***	-.021(.003)***	-.002(.011)	-.013(.013)	-.013(.012)
Activity Restrictions	.016(.018)	-.067(.040)**	-.006(.037)	-.399(.202)**	-.617(.202)***	-.386(.218)*
Deposit insurance	.164(.089)***	.685(.136)***	.466(.123)***	-1.079(.765)	-1.540(.82)*	-1.450(.898)*
AFC_dummy	-.782(.132)***	-491(.173)***	-.642(.128)***	-.834(.669)	.538(.846)	-1.129(.619)*
GFC_dummy	-.063(.071)	.062(.076)	-.043(.077)	.309(.430)	.387(.434)	.123(.402)
GDP growth	.019(.009)**	.032(.011)**	.023(.009)**	-.062(.037)*	.004(.054)	-.079(.046)*
Inflation	-.003(.007)	-.015(.007)***	-.008(.006)	-.138(.051)***	-.180(.049)***	-.159(.042)***
Constant	1.054(.388)***	2.421(.744)***	2.218(.591)***	25.06(4.210)***	28.49(4.727)***	30.03(5.557)***
Year dummy	Included	Included	Included	Included	Included	Included
No. of observation	1986	1986	1986	2060	2011	2060
No. of Banks	178	178	178	179	179	179
No. of instruments	177	177	177	178	178	178
Wald test (P-Value)	2009.92(0.00)	298.24(0.00)	347.19(0.00)	70.61(0.00)	89.28(0.00)	84.58 (0.00)
AR(1)(P-value)	-6.69(0.00)	-4.54(0.00)	-5.01(0.00)	-3.09(0.00)	-2.91(0.00)	-2.88 (0.00)
AR(2)(P-value)	1.072(0.284)	-0.50(0.62)	0.03(0.972)	-1.02(0.31)	-1.32(0.188)	-1.18 (0.237)
Hansen’J *x*^2^	164.72(0.47)	161.18(0.548)	159.51(0.584)	169.70(.364)	170.08(0.356)	169.10(0.376)
First Stage-F test (P-value)	24.7448 (0.00)	517.478(0.00)	22.3336(0.00)	25.86 (0.00)	507.106 (0.00)	24.63(0.00)
Wooldridge test (P-value)	177.259(0.00)	171.881(0.00)	168.157(0.00)	169.665(0.00)	172.158(0.00)	169.514(0.00)
Wu-Hausman test(P-value)	29.60(0.00)	62.78(0.00)	20.24(0.00)	52.89(0.00)	50.84(0.00)	46.60(0.00)
Breush–Pagan/Cook-Weisberg test (P-value)	129.43 (0.00)	140.61 (0.00)	123.24 (0.00)	1716.60 (0.00)	1721.11(0.00)	1866.58 (0.00)

Note: This table exhibits GMM regression outputs with robust standard error in order to correct heteroscedasticity among the banks. The dependent variable is lnZ-score based on ROAA in models 1–3 and equity ratio in models 4–6, as a proxy of financial soundness. H statistic, Lerner index, and HHI used as the measure of market power instrumented with property right and financial freedom. All regressors are listed and defined in S1 Table. Significant value of first stage regression and insignificant value of Hansen’s J test ensures that instrumental variable are relevant and valid. Significant value of Wooldridge test, Breush–Pagan /Cook-Weisberg test and Wu- Hausman test show there involves serial correlation, Heteroscedasticity and endogeneity and justify the use of GMM specification. Besides, significant value of Wald test implies that all models are correctly specified. The robust standard errors are reported in the parenthesis.

***, ** and * indicates the coefficient are significant at 1%, 5% and 10% significantly

**Table 5:** The effect of competition measured by H-statistic, Lerner index and HHI on NPL ratio as a measure of financial stability in ASEAN-5 from 1990–2014 period.

Dependent variable	NPL ratio		
Models	Model(1)	Model(2)	Model(3)
Lagged NPL ratio	.679(.059)***	.454(.068)***	.459(.069)***
H-statistic	-5.061(1.809)***		
H-statistic^2	3.586(1.640)**		
Lerner		.914(1.293)*	
Lerner^2		2.004(2.202)	
HHI			58.860(48.00)
HHI^2			-120.181(125.30)
Inflection Point	0.71	0.23	0.25
Sing of relationship	-	+	+
Loan to total assets	.109(.032)***	.085(.044)**	.099(.036)***
Bank size	-.660(.261)***	-1.463(.453)***	-1.617(.366)***
Foreign Ownership	.092(.653)	-.214(.969)	.362(.909)
Operational Efficiency	-.006(.015)	.033(.017)**	.040(.020)**
Activity restrictions	.875(.167)***	1.06(.236)***	.846(.261)***
Deposit Insurance	1.059(.861)	-.134(.835)	-.072(.775)
AFC_dummy	.655(.756)*	.199(.937)*	-.663(.933)
GFC_dummy	-.747(.410)*	-1.425(.455)***	-1.81(.530)***
Real GDP growth rate	-.262(.069)***	-.316(.078)***	-.396(.071)***
Inflation rate	.142(.047)***	.165(.067)***	.167(.060)***
Constant	-6.719(4.208)*	-1.915(4.758)	-4.385(5.205)
Year dummy	Included	Included	Included
No. of observations	2059	2059	2059
No. of Banks	179	179	179
No. of Instruments	121	178	178
Wald Test(P-value)	870.05(0.00)	483.11(0.00)	454.49(0.00)
AR(1) (P-value)	-3.93(0.00)	-3.59(0.00)	-3.69(0.00)
AR(2) (P-value)	-0.23(.822)	-0.74(0.460)	-0.78(0.433)
Hansen’J *x*^2^ (P-value)	125.47(0.171)	174.15(0.279)	168.95(0.379)
First Stage F test (P-value)	25.86(0.00)	507.10(0.00)	24.63(0.00)
Wooldridge test (P-value)	28.005(0.00)	28.658(0.00)	28.376(0.00)
Wu- Hausman test (P-value)	37.31(0.00)	7.7376(0.00)	35.84(0.00)
Breush–Pagan/Cook-Weisberg test (P-value)	1391.66 (0.00)	1270.36(0.00)	1349.96 (0.00)

Note: This table exhibits GMM regression outputs with robust standard error in order to correct heteroscedasticity among the banks. The dependent variable is NPL ratio as a proxy of credit risk. All regressors are listed and defined in S1 Table. H statistic, Lerner index, and HHI used as the measures of market power instrumented with property right and financial freedom. Significant value of first stage regression and insignificant value of Hansen’s J test ensures that instrumental variable are relevant and valid. Significant value of Wooldridge test, Breush–Pagan/Cook-Weisberg test and Wu- Hausman test show there involves serial correlation, heteroscedasticity and endogeneity respectively, and justify the use of GMM specification. Besides, significant value of Wald test implies that all models are correctly specified. The robust standard errors are reported in the parenthesis.

***, ** and * indicates the coefficient are significant at 1%, 5% and 10% significantly

In addition, the insignificant value of Hansen-J-test ensures the validity of overidentifying restrictions indicating that instrumental variables used for handling endogeneity problem are valid. That is, instruments are uncorrelated with error term and handled the endogeneity problem. Thus, heteroscedasticity problem is also handled, because, the presence of heteroscedasticity, overidentification restrictions would not be validated [64]. In addition, the significant value of AR (1) and insignificant value of AR(2) indicates that serial correlation is present at first order, but it is absent in the second order. Moreover, the significant value of Wald test implies that all models are correctly specified.

Model (1) of Table 4 demonstrates the effect of competition proxied by H-statistic along with control variables on *ln*Z-score. The results show that the coefficient of H-statistic is positive and significant, suggesting that any increase in the level of competition and/or decrease in the level of market power makes the ASEAN commercial banks more financially stable. Model (2) uses Lerner index as a measure of competition; the results show that the coefficient of Lerner index is negative and significant, indicating that any decrease in the level of market power or increase in the level of competition increases *ln*Z-score provided that higher value of Lerner index signifies less competition. Similarly, model (3) uses HHI as a measure of traditional competition through concentration. The sign of the coefficient of HHI is found negative but insignificant, meaning the traditional measure of competition through concentration is not a true measure for competition in ASEAN commercial bank. This finding is supported by Molyneux and Nguyen [65] and Liu, Molyneux [26] who also failed to find any significant relationship between concentration and risk taking of South-east Asian banks. The empirical work of Beck, Demirgüç-Kunt [66]; Moch [37] also consider concentration as an inappropriate measure of competition. This is because, the concentration ratio, which is based on Structure-Conduct-Performance (SCP) paradigm, suffers from both conceptual and practical limitations [67]. Under SCP paradigm, a rise in concentration is considered as rising collusive opportunities between banks which lead them to enjoy high market power and profitability. However, contestability theory of Baumol, Panzar [68] claims that a concentrated market can behave competitively if the barriers of entry and exist are lower. In this connection, Shaffer [69] suggests that anti-competitive behavior of the bank is not the result of structure rather due to conduct or efficiency. Bernheim and Whinston [70] also find that banks may also enjoy collusive opportunity in the presence of many firms. The weak applicability of the SCP paradigm in banking sector may be attributed to the different bank characteristics such as switching cost of retail borrowings, information asymmetries in corporate borrowings, and network externalities in the payment system [2].

Table 4 also investigates the effect of competition on the financial stability of commercial banks of ASEAN-5 considering equity ratio as a proxy of financial stability in models (4–6). The negative and significant coefficient of Lerner index in model (5) suggests that high competitive market induces banks with low market power to hold more equity capital which in turn makes them financially stable.

We also investigate the effect of competition on the financial stability of commercial banks in ASEAN-5 using NPL ratio as a measure of financial stability. The results are presented in Table 5. Model (1) of Table 5 exhibits the coefficient of H-statistic is negative and significant suggesting that any increase in competition and/or decrease in market power induces banks to take less credit risk in their loan portfolio. Again, model (2) shows that the coefficient of Lerner index is positive and significant which also indicates that high market power in the low competitive market motives the banks to take high credit risk. On the other hand, the coefficient of HHI in model (3) is insignificant which suggests that concentration has no effect on credit risk.

The overall results from Tables 4 and 5 suggest the financial sector should increase the level of competition in the banking market to enhance financial stability supporting the competition-stability view of Boyd and Nicolo [5]. Our results demonstrate that less market power in the highly competitive market leads the banks not only to hold more equity but also induces them to take less credit risk as well as overall risk in the financial market which ultimately increases their financial stability. These results suggest that financial stability of the banking sector in ASEAN-5 may channel through high capitalisation rate and low credit risk given a certain level of competition. The findings of the co-movement of capitalisation and financial stability support banking literature. For example, Allen, Carletti [71] predict that competition encourages banks to hold more equity capital, and this prediction is empirically supported by Schaeck and Cihák [31]. The reason of the co-movement of equity ratio and financial stability may be due to the fact that the capital buffer is built up through retained earnings, where, higher return on equity raises capitalisation as dividend pay-out ratio is comparatively fixed.

Our main results are consistent with earlier studies, which primarily focused on a particular geographical area, such as the European Union by Schaeck and Cihák [31], who also found that competition has a stability-enhancing effect by using a Boone indicator as a competition measure and the Z-score as stability during the period of 1995 to 2005. Further, the findings are also consistent with Asian emerging countries studied by Soedarmono, Machrouh[30], who also discovered similar findings using the Lerner index and efficiency-adjusted Lerner index as measures of competition, and the Z-score, standard deviation of ROA, and capitalisation ratio as stability measures during the period of 1994 to 2009. The results are also consistent with studies in broader areas, such as the work of Beck, Demirgüç-Kunt [66], covering 47 crisis episodes from 69 countries in the period of 1980–1997, which found that both concentration and competition have a stabilising effect on the banking system, while concentration alone is an insufficient measure of competitiveness. Anginer, Demirguc-Kunt [24] also found a negative relationship between systematic fragility and competition, measured by a Lerner index and H-statistic, on 1,942 banks from 68 counties during 1998 to 2010.

The results also contradict earlier studies focusing on a particular geographical area, such as Latin American countries by Yeyati and Micco [21], who found a fragility effect of competition during 1993 to 2002, capturing risk-taking by the Z-score and competition by H-statistic. Further, these are also different from the findings of Fu, Lin [9], who focused on the Asia-Pacific region. This study found that low market power, measured with the Lerner index, induces high-risk exposure, measured with both the Z-score and probability of default approach from the work of Merton [25] for both listed and non-listed banks, but concentration significantly fostered financial fragility during 2003 to 2010. However, low-risk profiling of the banks with low market power in competitive commercial banking market in ASEAN-5 is not surprising. Because, Aftermath of the AFC in 1997–98 when all ASEAN-5 banks suffered from substantial capital erosions and bank failures at different levels, the banking sector has gone through tremendous restructuring process including consolidation in the form of merger and acquisition, widening the score of foreign ownership in the domestic banking market, regulatory reform in the form of capital regulation, market discipline and supervision [72–74]. The post-crisis restructuring, deregulations and supervisory drives resulted in strengthening capital base, risk management capability and earning capability (See S2 Table) which may enhance the financial stability in the region.

The aforementioned quadratic term of competition measures are also used in our model following the work of Berger, Klapper[18], Tabak, Fazio [75], and Fu, Lin [9] to find the non-linearity between competition and stability in ASEAN-5 countries during 1990 to 2014. Model (1) of Table 4 demonstrates a negative and significant sign of the quadratic term of H-statistic regressed on *ln*Z-score given a positive and significant of the linear term of H-statistic. This suggests that the relationship between competition and financial stability is non-linear and inverted U-shaped. Inflection points are calculated for the variables of interest, and are compared with the data set to understand the relationship between competition and financial stability. The inflection point in model (1) is greatest at +0.66, which is the approximate 75^th^ percentile, indicating that 75% of the data in the H-statistic distribution lies below the inflection point. Likewise, model (1) of Table 5 also exhibits a positive coefficient of the quadratic term of H-statistic regressed on NPL ratio, given a negative coefficient on the linear term, also suggesting a non-linear relationship between competition and financial stability. Here, the inflection point is + 0.71, which is also the approximate 75^th^ percentile, also indicating 75% of the data in the H-statistic distribution lies below the inflection point.

First, among the control variables, predictably, efficient banks are found to be more financially sound and less prone to both credit risk and overall risk, though they hold less capital than inefficient banks in ASEAN-5. Second, large banks are more financially sound and less sensitive to credit risk, despite keeping less capital in comparison to small banks; this may be due to efficiency gain through large ASEAN-5 banks’ economic scale benefits. Third, the level of intermediation, or the size of the net loan in comparison to total assets, reduces capitalisation and increases the level of credit risk; unexpectedly, this also enhances ASEAN-5 banks’ financial soundness. This may be due to the utilisation of high growth prospects in emerging ASEAN-5 countries. Although foreign banks are found to be more capitalised, no significant evidence indicates that they are less prone to credit risk and a high tendency towards financial soundness in comparison to domestic banks. This may be due to stricter capital regulations and less portfolio diversification.

Regarding the financial crises, the effect of the 1997–1998 AFC is much more severe and intensive for ASEAN-5 banks than the 2008–2009 GFC. During the AFC, ASEAN banks took excessive risk and lost their capitalisation to a greater extent, which made them fragile from losing franchise value and facing higher moral hazards in the form of gambling for resurrection and looting [20]. Conversely, during the GFC, ASEAN-5 banks were less resilient to risk-taking initiatives due to their high capitalisation and efficient loan portfolio management, based on the experiences of the preceding AFC.

Beck, De Jonghe [47] argue that regulatory framework may alter the competition-stability nexus; therefore, this study also controlled for activity restrictions and deposit insurance. It is found that activity restrictions encourage banks to be less capitalised and to become more involved in risk-taking initiatives if their finding of diversified clients or open business lines is restricted. This finding is consistent with Anginer, Demirguc-Kunt [24], who advocated for removing activity restrictions to foster competition. However, explicit deposit insurance has a significantly stabilising effect in ASEAN banks by protecting banks from a run, as suggested by Diamond and Dybvig [12]. Among the country-level control variables, inflation has a fragility effect by increasing risk-taking initiatives, and real GDP growth has a stabilisation effect by reducing risk-taking initiatives.

### Robustness checking

A number of robustness checks were conducted for our main results. Firstly, we re-estimated our main results eliminating quadratic term of competition following the works of Fu, Lin [9] and Kasman and Kasman [27]. The results are reported in S3 Table. The results demonstrate that our main findings of financial stability effect of competition are robust even in absent of quadratic term of competition measure. In the main models, we used dynamic panel model in examining the competition-stability nexus. Here we also run static fixed effect and/or random effect model based on Hausman test results following the works of Agoraki, Delis [10], Beck, De Jonghe [47] and Anginer, Demirguc-Kunt [24], and the results are reported in S4 Table. We also have found that the competition increases financial stability in the banking sector in static models as well. In addition, instead of HHI as a measure of concentration, we used large three banks market share in loan market (CR3), and also have found the similar evidence. The coefficient of CR3 also is found negative and insignificant for *ln*Z-score as a measure of financial stability. This result is not reported here, but this is available if required. Thus, the results robustly confirm the findings that competition is supportive for the financial stability in ASEAN banking sector.

## Conclusion

This study investigates the nexus between competition and financial stability using an unbalanced panel data from commercial banks of ASEAN-5 countries over the 1990–2014 period. Three measures of financial stability were used, namely the natural logarithm of Z-score to proxy bank stability, NPL ratio to proxy loan portfolio risk and equity ratio to proxy capitalisation. Meanwhile, competition was measured through both structural approach (Herfindahl–Hirschman index) and non-structural approaches (Panzar-Rosse H-statistic and Lerner index) and regressed them separately on financial stability models.

The results show that H-statistic has a positive and significant effect on *ln*Z-score, and Lerner index has a negative and significant effect on *ln*Z-score, suggesting that increases in the level of competition and decreases in market power promote the financial stability in the banking sector. Again, the specification where equity ratio was used as dependent variable, the results indicate that Lerner index has a negative and significant effect on equity ratio, suggesting that decreases in market power stimulates banks to hold more equity capital which promotes financial stability. We also used NPL ratio as dependent variable, the results exhibit that H-statistic has a negative and significant effect on NPL ratio and Lerner index has a positive and significant effect on NPL ratio, suggesting that increases in the level of competition and decreases in the level of market power induce banks to take less credit risk which ultimately promotes financial soundness of the sector. The specifications where HHI was used as competition proxy, the results do not show any significant effect of HHI on neither of *ln*Z-score, equity ratio and NPL ratio, suggesting that bank concentration has no effect on financial stability in this sector.

To capture the non-linear relationship between competition and financial stability, we included a quadratic term of competition measures (H-statistic, Lerner and HHI) in our specifications. The results indicate that square term of H-statistic has a negative and significant effect on *ln*Z-score, given a positive and significant effect in the linear term. Also, in another specification where NPL ratio was used as dependent variable, the results show that square term of H-statistic has a positive and significant effect on NPL ratio, given a negative and significant effect in the linear term. These results suggest that the relationship between competition and financial stability is non-linear or inverted U-shaped which may imply that the margin effect (competition-fragility view) starts dominating over the risk-shifting effect (competition-stability view) after reaching the inflection point.

In our specification, we included two crisis dummies to capture the 1997–98 AFC and 2008–09 GFC, to determine the effect of the financial crisis on the financial stability of the banking sector. The results indicate that AFC has a negative and significant effect on both *ln*Z-score and equity ratio and positive and significant effect on NPL ratio, suggesting that AFC eroded capitalisation and increased credit risk which undermines the financial soundness of the banking sector.

The overall results indicate that competition (measured by H-statistic) is positively related to financial stability and capitalisation (measured by *ln*Z-score and equity ratio respectively), and negatively related to credit risk (measured by NPL ratio). Also, market power (measured by Lerner index) negatively related to financial stability and capitalisation, and positively related to credit risk. These results demonstrate that increase in competition and decrease in market power influence banks to hold more capital and take less credit risk which enhance their financial stability. This evidence strongly supports the competition-stability view of Boyd and Nicolo (5) for the commercial banks in ASEAN-5. The results also clarify a non-linear or inverted U-shaped relationship between competition and financial stability in the region, supporting the neutral view of Martinez-Miera and Repullo [6]. The results also indicate that the traditional measure of competition through concentration ratio is insufficient in explaining the effect of competition on financial stability in the ASEAN-5 market.

Additionally, the results find that large banks are more efficient, and their higher level of intermediation enhances bank stability. Banks were found to be highly volatile and lost capitalisation during the AFC, as expected, but the GFC did not have as much of an impact due to superior loan portfolio quality and capitalisation. Although explicit deposit insurance as regulatory instrument enhances financial stability, stricter regulatory restrictions weaken it, limiting banks’ scope to enhance their portfolio.

The findings lead to policy recommendations for policy makers of ASEAN banks, to enhance financial stability and the successful implementation of the ASEAN Banking Integration Framework, or ABIF. As concentration is insignificant to financial stability, consolidation may be an inappropriate policy for reducing competition in the banking sector. Rather, competition is increased due to the liberalisation of restrictions on foreign banks. As activity restrictions demonstrate high sensitivity to banks’ risk-taking initiatives, appropriate actions may be taken to increase the scope of banking operations, to enhance competition. Deposit insurance has a stability-enhancing effect and could be more explicit in the region. It is found that large banks are less capitalised in a competitive market in the presence of deposit insurance; hence, capital regulations should be stricter to offset the negative effects of capital shortage. Appropriate policies should be enacted to foster competition, which has a regional stabilising effect, as well as the efficiency of the banking system, by taking care of large banks in the form of a “too-big-to-fail” policy. This is because large banks may have a contagion effect on the entire market, and the failure of a large bank may make the whole banking market fragile, as banks are interconnected.

Our work provides stimulus to compute the stability measures for the banks that failed just before they failed. It further stimulates for a disaggregate analysis of the role of bank regulation, particularly capital requirements, deposit insurance and activity restrictions in shaping financial stability through the channel of competition. This helps to determine how and which regulation works well in promoting financial stability through the channel of competition.

## Supporting information

S1 Table Variable definition and sources of data collection. Note: ^a^Expected sign is assigned if dependent variable is either Z-score or equity ratio. A reverse sign is expected if dependent variable is NPL ratio.
(PDF)

S2 Table Capitalization, risk management capacity and earning capacity of each ASEAN-5 countries in 1998, 2008, and 2014. Note: Data source is Bankscope database of BVD; Capitalization is measured with the ratio of equity on total assets, assets quality is measured with the ratio of non-performing loan on gross loan and earning capacity is measured with the ratio of return on average total assets.
(PDF)

S3 Table The effect of competition measured by H-statistic, Lerner index and HHI on Z-score and equity ratio and NPL ratio as measures of financial Stability. Note: This table exhibits GMM regression output with robust standard error in order to correct heteroscedasticity among the banks. The dependent variable is lnZ-score based on ROAA in models 1–3 and equity ratio from models 4–6 as a proxy of financial soundness. H statistic, Lerner index, and HHI used as the measure of market power instrumented with property right and financial freedom. All regressors are listed and defined in S1 Appendix. The robust standard errors are reported in the parenthesis. ***, ** and * indicates the coefficient are significant at 1%, 5% and 10% significantly.
(PDF)

S4 Table The effect of competition measured by H-statistic, Lerner index and HHI on Z-score and equity ratio and NPL ratio as measures of financial Stability in static models. Note: This table exhibit Fixed effect regression output with robust standard error in order to correct heteroscedasticity among the banks. The dependent variable is lnZ-score based on ROAA in models 1–3 and equity ratio from models 4–6 as a proxy of financial soundness. H statistic, Lerner index, and HHI used as the measure of market power instrumented with property right and financial freedom. All regressors are listed and defined in S1 Appendix. The robust standard errors are reported in the parenthesis.
***, ** and * indicates the coefficient are significant at 1%, 5% and 10% significantly.
(PDF)
