# Supplementary material for: Does competition improve financial stability of the banking sector in ASEAN countries? An empirical analysis
Source: PLoS One. 2017 May 9;12(5):e0176546. doi: 10.1371/journal.pone.0176546 (PMC5423589; doi:10.1371/journal.pone.0176546)
Supplement: S1 Table — Note: aExpected sign is assigned if dependent variable is either Z-score or equity ratio. A reverse sign is expected if dependent variable is NPL ratio. (PDF) [file pone.0176546.s001.pdf]

Variable	Definition and data source	Expected sign[a]
**Dependent variables**		
**Z-score**	Z-score is a measure of bank's soundness. Higher value indicates higher stability and higher distance from probability of default. We use natural logarithm of Z-score in order to normalize the data. Data source: Bankscope database Bureau Van Dijk (BVD)	
**NPL ratio**	A ratio of Non-performing loan to gross loan, high value indicates higher credit risk and more risky loan portfolio. Data source: Bankscope database of BVD	
**Equity ratio**	A ratio of equity to total assets, high value indicates lower loan portfolio risk and better capitalization. Data source: Bankscope database of BVD	
**Variables Measuring competition and concentration**		
**H-statistic**	Non-structural measure of competition which is the sum of elasticity of revenue with respect to input prices. Higher value of H-statistics indicates higher competition and lower market power. Data source: Bankscope database of BVD	+
**Lerner Index**	Non-structural measure of competition which is a mark-up of price over marginal cost. Higher value indicates less competition and higher market power to set loan price over marginal cost. Data source: Bankscope database of BVD	-
**HHI**	Structural measure of competition which is the sum of square of market share of loan of commercial banks. Higher value indicates high concentration and market power, and low competition. Data source: Bankscope database of BVD	-
**Bank Level Variables**		
**Bank size**	Log of total assets in million US$. Data source: Bankscope database of BVD	+
**Assets composition**	Ratio of net loan to total assets indicating size of intermediation or asset composition. Data source: Bankscope database of BVD	-
**Operational Efficiency**	The ratio of cost to income is used to proxy bank efficiency. Data source: Bankscope database of BVD	-
**Foreign Ownership**	A dummy variable having value 1 if foreign ownership exceeds 50 % of total ownership. Data source: Bankscope database of BVD and bank's website	+
**Regulation variables**		
**Deposit Insurance**	A dummy variable which takes 1 for the country having explicit deposit insurance scheme otherwise 0. Data source: Barth et al.(2000,2003,2008)	+
**Activity Restrictions index**	An index that takes a value from 0 to 12. Higher value shows greater regulatory restriction on bank activities. Data source: Barth et al.(2000,2003,2008)	-
**Macro-economic Variables**		
**Inflation rate**	Annual Inflation rate based on consumer price index. Data source: World bank development indicator. Data source: World Bank development indicator	-
**Real GDP growth rate**	Annual real GDP growth rate. Data source: World bank development indicator	+
**Asian Crisis dummy**	A dummy variable that takes 1 if the year is 1997 to 1998, otherwise 0. Data source: Authors' compilation	-
**Global Crisis dummy**	A dummy variable that takes 1 if the year is 2008 to 2009, otherwise 0. Data source: Authors' compilation.	-
**Instrumental variables**		
**Financial Freedom**	An index that takes value from 0-100 indicating the level of regulatory restrictions on financial freedom of the firms. Higher value indicates more freedom and less restrictions. Data source: Heritage Foundation	
**Property Right**	An index that takes a value from 0 -100 indicating the level to which private property right is protected by the law. A higher score indicates more economic freedom and strong protection of property right of the individuals. Data source: Heritage Foundation	
