# Supplementary material for: Does competition improve financial stability of the banking sector in ASEAN countries? An empirical analysis
Source: PLoS One. 2017 May 9;12(5):e0176546. doi: 10.1371/journal.pone.0176546 (PMC5423589; doi:10.1371/journal.pone.0176546)
Supplement: S2 Table — Note: Data source is Bankscope database of BVD; Capitalization is measured with the ratio of equity on total assets, assets quality is measured with the ratio of non-performing loan on gross loan and earning capacity is measured with the ratio of return on average total assets. (PDF) [file pone.0176546.s002.pdf]

	Indonesia			Malaysia			Philippines			Singapore			Thailand		
Years	1998	2008	2014	1998	2008	2014	1998	2008	2014	1998	2008	2014	1998	2008	2014
Capitalization	-3.70	13.25	13.27	9.75	10.77	10.89	18.25	12.83	14.26	24.88	23.29	10.90	5.81	10.96	11.81
Risk Management Capacity	36.46	3.58	3.10	11.13	2.06	1.26	11.88	8.78	8.37	7.00	1.89	.97	48.46	7.03	3.05
Earning capacity	-15.27	0.130	1.23	0.32	1.30	1.0	1.23	.661	1.02	.62	.867	.76	-7.14	.60	1.07
