# Supplementary material for: Does competition improve financial stability of the banking sector in ASEAN countries? An empirical analysis
Source: PLoS One. 2017 May 9;12(5):e0176546. doi: 10.1371/journal.pone.0176546 (PMC5423589; doi:10.1371/journal.pone.0176546)
Supplement: S4 Table — Note: This table exhibit Fixed effect regression output with robust standard error in order to correct heteroscedasticity among the banks. The dependent variable is lnZ-score based on ROAA in models 1–3 and equity ratio from models 4–6 as a proxy of financial soundness. H statistic, Lerner index, and HHI used as the measure of market power instrumented with property right and financial freedom. All regressors are listed and defined in S1 Appendix. The robust standard errors are reported in the parenthesis. ***, ** and * indicates the coefficient are significant at 1%, 5% and 10% significantly. (PDF) [file pone.0176546.s004.pdf]

Dependent variable	*ln*Z-score			Equity ratio			NPL ratio		
Model and measures of competition	Model-1 H-Stat.	Model-2 Lerner Index	Model-3 HHI (loan)	Model-4 H-stat.	Model-5 Lerner Index	Model-6 HHI (loan)	Model-7 H-stat.	Model-8 Lerner Index	Model-9 HHI (loan)
Competition	2.0913	-.7303	5.1970	-13.0165	2.0121	-141.569	1.5049	.7624	-49.3993
	(.251)***	(.129)***	(3.366)	(1.837)***	(.9531)**	(24.316)	(1.233)	(.628)	(16.224)
Competition^2	-1.8090	.0542	-10.8137	8.3469	.7043	178.4239	-.7316	1.2656	74.5777
	(.209)***	(.255)	(7.532)	(1.529)***	(1.867)	(54.634)	(1.028)	(1.232)	(36.456)*
Loan to Assets	-1.809	.0149	.0170	-.0315	-.0452	-.0276	-.0704	-.047	-.0464
	(.209)***	(.002)***	(.002)***	(.014)**	(.014)***	(.014)**	(.009)***	(.010)***	(.0093)***
Bank Size	.0997	.1350	.1198	-3.1868	-3.3292	-3.9423	-3.021	-3.865	-3.9958
	(.042)***	(.042)***	(.043)***	(.309)***	(.312)***	(.312)***	(.165)***	(.206)***	(.208)***
Operational Efficiency	-.0526	-.0068	-.0066	.0078	.0109	.0092	-.003	-.0016	-.0023
	(.050)***	(.001)***	(.001)***	(.004)*	(.004)**	(.004)**	(.003)	(.003)	(.003)
Foreign Ownership	-.0059	-.0486	-.0552	.9115	1.0106	.7313	.129	.0696	.0058
	(.001)	(.051)	(.051)	(.371)**	(.375)***	(.371)***	(.251)	(.248)	(.247)
Activity Restrictions	-.2749	-.4130	-.3577	.9704	1.1393	2.2774	-1.433	-2.1438	-1.9249
	(.033)***	(.035) ***	(.037)***	(.243)***	(.257)***	(.263)**	(.134)***	(.170)***	(.175)***
Deposit insurance	.5779	.8840	.6153	3.904	4.4169	4.0450	-.5444	.6100	1.0764
	(.091)***	(.106) ***	(.093)***	(.672)***	(.788)***	(.673)***	(.415)	(.520)	(.449)**
AFC_dummy	-.0853	.1222	.0218	1.2442	.9169	.1546	.5634	.9135	.8551
	(.109)	(.111)	(.111)	(.805)	(.818)	(.798)	(.541)	(.539)	(.532)
GFC_dummy	-.077	.0224	-.0348	-2.3156	-2.8705	-2.726	-.2629	-.4478	-.3323
	(.085)	(.084)	(.085)	(.632)***	(.632)***	(.621)***	(.426)	(.416)	(.414)
Real GDP growth	.0434	.0643	.0551	-.4784	-.6207	-.504	-.0145	.0364	.0778
	(.010)***	(.010)***	(.010)***	(.074)***	(.072)***	(.072)***	(.049)	(.047)	(.048)*
Inflation	.0064	.0032	.0086	-.0204	-.0794	.0484	-.1946	-.1619	-.1341
	(.005)	(.005)	(.005)	(.036)	(.037)**	(.038)	(.023)***	(.024)***	(.025)***
Constant	4.2328	5.8599	4.8959	26.3422	23.0475	27.7751	56.0511	68.4297	71.4802
	(.561)***	(.570)***	(.583)***	(4.136)***	(4.196) ***	(4.212) ***	(2.229) ***	(2.767) ***	(2.810)***
Year dummy	Yes	Yes	Yes	Yes	Yes	Yes	Yes	Yes	Yes
No. of Observations	2394	2394	2394	2431	2431	2431	2432	2432	2432
No. of Banks	179	179	179	179	179	179	179	179	179
R^2	0.2384	0.226	0.2131	0.2346	0.2173	0.2397	0.2276	0.1986	0.2032
F test (P-value)	57.47	53.72	49.71	57.21	51.83	58.86	553.36	46.29	47.62
	(0.00)	(0.00)	(0.00)	(0.000	(0.00)	(0.00)	(0.00)	(0.00)	(0.00)
Hausman test (Chi^2)	-125	-223.58	-298.85	-104.76	-31.36	-40.99	4.88	-80.80	-115.28
(P-value)	(0.00)	(0.00)	(0.00)	(0.00)	(0.00)	(0.00)	(.9618)	(0.00)	(0.00)
